# Survey Research Among Older Migrants: Age-Related Differences in Contact and Cooperation

**DOI:** 10.1093/geront/gnac017

**Published:** 2022-01-29

**Authors:** Verena Seibel, Marieke Haan

**Affiliations:** Department of Interdisciplinary Social Sciences, Utrecht University, Utrecht, The Netherlands; Sociology Department, University of Groningen, Groningen, The Netherlands

**Keywords:** Incentives, Mode choice, Response rate

## Abstract

**Background and Objectives:**

Given the increasing academic interest in older migrants, this study aims at examining the likelihood of establishing contact and cooperation in a survey among first-generation migrants in Germany, comparing migrants of age 50 and older with younger migrants (aged 16–49).

**Research Design and Methods:**

We analyze data from the Migrants’ Welfare State Attitudes (MIFARE) study collected in Germany, which contains information about first-generation migrants from 9 different origin groups living in private households. Potential survey participants were contacted via mail and invited to participate in a paper- or online-based survey. In addition, an incentive experiment was implemented and age-dependent response rates were analyzed. Using logistic regression analyses, we compare older and younger migrants with regard to their likelihood of contact, cooperation, reaction to incentives, and mode choice.

**Results:**

Within the MIFARE study, older migrants are more likely to be contacted than younger ones. Older migrants are also more likely to cooperate in survey research than younger migrants. Both groups respond equally positively to the use of unconditional incentives. Lastly, older migrants show a strong preference to fill out the questionnaire on paper, rather than online.

**Discussion and Implications:**

Older first-generation migrants living in private households are easier to contact and are more likely to cooperate in survey research than younger first-generation migrants. Offering unconditional incentives and surveys on paper are likely to increase response rates among older migrants.

Research in the field of migration shows an increasing interest in older migrants, particularly in the fields of health care and well-being (Ciobanu & Hunter, 2017; Ten Kate et al., 2020; Vaillant & Wolff, 2010; Van Tilburg & Fokkema, 2018; Warnes & Williams, 2006). Older migrants differ from younger migrants in their migration history, integration trajectories, and health status; factors that make them particularly interesting to study, but which also are likely to affect their likelihood to participate in research. For researchers in the field of quantitative research, the challenge arises how to acquire a representative sample of the older migrant population for survey research. We know from previous studies that migrants are not only more difficult to contact than natives (Feskens et al., 2006; Haan et al., 2014), they also show significantly lower cooperation rates, meaning that they are less likely to be able and/or willing to participate in research (Feskens et al., 2006). Although several studies have examined main factors determining migrants’ contact and cooperation probabilities (Deding et al., 2008; Feskens et al., 2006; Fick & Diehl, 2013; Kappelhof, 2014), we know very little about how these factors relate to older migrants in particular (Sin, 2004). Using the Cost–Benefit Theory (Singer, 2011), the Leverage–Saliency Theory (Groves et al., 2000), and the Social Isolation Theory (Deding et al., 2008), we provide several reasons to believe that older migrants might behave differently in the survey field than younger migrants. Reasons include different language barriers, different mobility behavior, or different attitudes toward surveys (Deding et al., 2008; Schmid & Keijzer, 2009; Ten Kate et al., 2020).

In this article, we therefore contribute to the current debate on how to target older migrants in large-scale surveys by analyzing response rates among nine different migrant groups, comparing older migrants aged 50–75 to migrants aged 16–49. We aim at answering the following research questions: First, do older migrants differ from younger migrants in their likelihood of being contacted, hence, receiving an invitation to the survey? Second, among those who have been contacted, do older migrants differ from younger migrants in their likelihood to participate in the survey? The third research question is related to the second: to what extent do different types of incentives influence the response behavior of older migrants compared with younger migrants? And fourth, among those who have participated in the research, do older migrants differ from younger ones in their preference for mode of questionnaires (paper vs. online)?

We use data from the project Migrants’ Welfare State Attitudes (MIFARE), which contains information on contact and cooperation and mode choice among first-generation migrant groups. In addition, the data contain an incentive experiment (Bekhuis et al., 2018) that allows us to examine whether older migrants respond differently to incentives than younger migrants. In the following, we first discuss possible age differences in the likelihood of contact. We then continue with a discussion on age differences among migrants regarding their likelihood of cooperation. In this section, we also discuss possible differences between older and younger migrants in their reaction toward incentives as well as their preferences for paper-based versus online questionnaires. After describing the data and research design, we continue with logistic analyses. We thereby conduct several robustness checks to test whether the results hold equally for older male and female migrants and for different migrant groups. We end this article with a discussion of the results and possible implications for future research.

## The Likelihood to Contact Older Migrants

Next to defining the target population (in this study: first-generation migrants from nine different origin groups) and deciding on a sample frame (in this study: resident registers on the municipality level), researchers are confronted with the challenge of contacting potential survey participants. Contact can occur via telephone, written invitation via post, and invitation via email, with each contact mode having its advantages and disadvantages. Contact via telephone has become increasingly difficult, mainly because of the rising numbers of nonregistered cell phone numbers, particularly among migrant populations (Feskens et al., 2006; Granato, 2010). Research also finds significantly lower response rates among surveys distributed via email than via post (Bech & Kristensen, 2009; Daikeler et al., 2020; Shih & Fan, 2008). In particular, older migrants are less likely to hold an email account (Millard et al., 2018), which can lead to an age-related selection bias. The MIFARE researchers therefore chose a written invitation letter, followed by two reminders (Bekhuis et al., 2018). However, when contacting migrants for survey participation via post, researchers must consider their mobility. Migrants are found to move more often between addresses within the host country than natives and are, by definition, more likely to move out of the country than natives (Blohm & Diehl, 2001).

Age plays a role regarding mobility patterns. For older migrants, mobility is often connected to returning to their home country when reaching retirement age (Cobb-Clark & Stillman, 2013). However, research suggests that postretirement migration flows are rather small (Rallu, 2017). One of the main reasons for older migrants to stay in the receiving country, despite different initial anticipations, is the core family that continues to live in the host country. Older migrants tend to stay in the receiving country the longer they have lived in the receiving country, and when they have children living in the receiving country, which applies to most older migrants. Although older migrants tend to visit their home country, research shows that older migrants are significantly less mobile than younger migrants (Ciobanu & Hunter, 2017; Clark & Drever, 2000). Like younger natives, younger migrants are often affected by mobility-enhancing factors such as family formation or job opportunities, which affect older migrants to a lesser extent (Ciobanu & Hunter, 2017). This is in line with previous research on general populations showing that contact probability is higher among older potential respondents than among younger potential respondents (Stoop, 2005). Hence, we formulate our first hypothesis:

H1: Older migrants are more likely to be contacted than younger migrants.

## Likelihood of Cooperation

After potential participants have been contacted, the question arises whether they cooperate, that is, participate in the survey. Most surveys deal with significant nonresponse rates due to people’s inability or unwillingness to participate (Haan & Ongena, 2014). There are mixed debates about whether older people are more or less likely to participate in survey research. According to the Cost–Benefit Theory (Singer, 2011), people participate in a survey if the benefits of participation outweigh the costs of participation. The ability and willingness to participate in survey research depend also on people’s health (Gaertner et al., 2016). Because the occurrence of physical and mental health problems increases with age, older people might perceive survey participation more burdensome and costly than younger people, thereby reducing their willingness and ability to participate in survey research (Gaertner et al., 2016; Motel-Klingebiel et al., 2019). In addition, a significant number of migrants are not fluent in the host country’s language. Low language skills increase the costs of survey participation tremendously and nonresponse among migrants is often due to these language problems (Feskens et al., 2010). This might be particularly true for older migrants who either migrated during a time when language courses were not offered to the extent they are now (Danzer & Yaman, 2016), or because of language attrition, in which the host country language recedes in age and the mother tongue gains in importance (Schmid & Keijzer, 2009). Previous research shows indeed that lack of cooperation among older migrants is often due to their lower host language skills (Feskens et al., 2006). However, within the MIFARE project, invitation letters, reminders, and questionnaires were offered in two languages: German and the main language of the home country. Hence, language barriers are not likely to play a role in older migrants’ cooperation behavior within this project.

Next to the Cost–Benefit Theory, the Social Isolation Theory predicts that people who are socially isolated (e.g., people who have a small network and/or no employment) have lower cooperation rates (Deding et al., 2008). Studies indicate that, though having social contacts, older migrants report high levels of loneliness (Cela & Fokkema, 2017; Ten Kate et al., 2020). This subjective feeling of loneliness is likely to contribute to older migrants’ perceived social isolation and disengagement from society; factors that are negatively associated with cooperative behavior within survey research (Deding et al., 2008).

Research among native populations shows that the likelihood of cooperating in mail surveys decreases with age (Groves & Couper, 2012; Kaldenberg et al., 1994). However, there are also good reasons to believe that cooperation is higher among older than younger migrants (Lusk et al., 2007). According to the Leverage–Salience Theory (Groves et al., 2000), survey cooperation increases with a higher sense of civic duty. Indeed, older age is associated with a higher sense of civic duty, which is likely to increase the cooperation rate (Deding et al., 2008). Results on willingness to cooperate are indeed mixed. A survey among migrants in Denmark has not found an effect of age on the likelihood of cooperation in surveys, although their sample included only first-generation migrants until the age of 45 and nothing can therefore be said about first-generation migrants older than the age of 50 (Deding et al., 2008). Kappelhof (2014) finds an underrepresentation of younger first-generation migrants among Moroccan samples in the Netherlands, suggesting higher response rates among older first-generation migrants than younger first-generation migrants. Given the mixed results on the effect of age on cooperation behavior, we formulate two contradicting hypotheses:

H2a: Older migrants are less likely to cooperate in surveys than younger migrants.

H2b: Older migrants are more likely to cooperate in surveys than younger migrants.

Last, but not least, incentives are frequently used to boost response rates. Research generally shows that unconditional incentives are associated with higher cooperation rates than are conditional incentives, particularly for mail surveys (Mercer et al., 2015). However, conditional incentives should not be too high in order not to overshadow peoples’ intrinsic motivation (Becker et al., 2007). Most incentive experiments applied in survey research use monetary incentives for both the conditional and unconditional incentives (Fick & Diehl, 2013; Singer, 2018; Stadtmüller & Porst, 2005). This was not the case in the MIFARE project. Survey participants were randomly distributed across four experimental conditions: Group 1 received an unconditional incentive only (small screen cleaner for mobile phones with the logo of the University of Konstanz, delivered with the first invitation letter); Group 2 was offered a conditional incentive (€10 voucher from Amazon, Media Markt, or Mueller), which was provided after participation in the survey; Group 3 was offered both the unconditional and the conditional incentives; and Group 4 did not receive any incentive at all. Given that in the MIFARE project, the conditional and unconditional incentives vary in their nature (monetary vs. nonmonetary), we must be careful with generalizing results from previous studies, which usually use monetary incentives in all their conditions.

To our knowledge, research about the use of incentives among older migrants is missing. Based on previous research, however, we expect that it is likely that older migrants respond more strongly to a monetary conditional incentive than to a nonmonetary unconditional incentive. First, older migrants face a higher risk of age-related poverty (Mika & Tucci, 2006), which creates an incentive to participate in surveys if vouchers are provided. Hence, according to the Cost–Benefit Theory (Singer, 2011), monetary vouchers increase the benefits of survey participation. Second, the unconditional incentive that incorporates the University logo might create an artificial distance between the survey organization and older migrants. Older migrants are less likely to identify with the receiving country and might therefore perceive its researchers more as an outgroup (Fick & Diehl, 2013; Johnson et al., 2002). We therefore hypothesize that:

H3a: The conditional incentive (voucher) increases response rates among older migrants more strongly than among younger migrants.

H3b: The conditional incentive (voucher) increases response rates among older migrants more strongly than the unconditional incentive (mobile screen cleaner).

Last, but not least, we were interested in whether older migrants, once agreeing to participate in the survey, show preferences for specific survey modes. Previous research has shown that survey participants have preferences for different modes of responding (Haan et al., 2014). To increase sample members’ willingness to participate in the survey, the MIFARE survey offered a concurrent design of mode choice, meaning that “sample members are offered a choice of modes during first contact” (Haan et al., 2014). The MIFARE design offers two modes of participation: online and paper-based participation. Both options were offered simultaneously, and survey participants could choose depending on their individual preferences. Web-based surveys are connected to respondents’ digital skills, which are generally lower among the older population (Millard et al., 2018). Hence, the costs of participating in online surveys are higher for older migrants than the participation in paper-based surveys. Research among general populations shows that indeed, older people prefer nonweb response modes (Miller et al., 2002). In addition, previous studies suggest that whereas ethnic minorities seem to prefer participating in postal surveys, web-based surveys are more successful among majority members (Schneider et al., 2005). We therefore hypothesize that:

H4: Older migrants are more likely to opt for paper-based questionnaires than are younger migrants.

## Data, Measurements, and Methods

### Data Collection

To answer our research questions and test our hypotheses, we make use of the German data from the survey MIFARE. The MIFARE data were collected in the years 2015–2016 among nine different migrant groups from Eastern Europe (Russia, Poland, and Romania), Western Europe (Great Britain, Spain, and the United States), Asia (Japan and China), and Turkey. All migrants surveyed were born in their country of origin and migrated to Germany after the age of 16. Moreover, survey participants were between the age of 16 and 75 at the time of the survey (Bekhuis et al., 2018). Representative samples were drawn from 40 municipalities based on the distribution of these migrant groups in Germany. As mentioned before, potential survey participants were approached with a written invitation letter, both in German and in the main language of their origin country, containing the questionnaire as well as a link to a webpage, where the survey could be filled out online. Moreover, potential participants had the choice to answer the questionnaire either in the main language of their country of origin or in German. This provided all migrants (who were literate at least in the main language of their country of origin) the opportunity to participate in the survey. For this study, we compare the contact rate and cooperation rate of two age groups: migrants between the age of 16 and 49 (here: younger migrants) and migrants between the age of 50 and 75 (here: older migrants). Migrants older than the age of 50 have a significantly lower likelihood of participating in the labor market, which is one of the main factors explaining response behavior (Ciobanu & Hunter, 2017).

### Measurements

The *likelihood of contact* was measured by subtracting the number of invitation letters that could not be delivered to the respective addresses from the number of total invitation letters sent to potential survey participants. Nondelivery was observed if the invitation letter and/or reminder was sent back to the sender because the survey respondent was not living at the given address anymore. Survey participants were randomly distributed across four experimental *incentive groups*: Group 1 received an unconditional incentive only (small mobile screen cleaner with the logo of the University of Konstanz, delivered with the first invitation letter); Group 2 was offered a conditional incentive (€10 voucher from Amazon, Media Markt, or Mueller), which was provided after participation in the survey; Group 3 was offered both, the unconditional and the conditional incentives; and Group 4 did not receive any incentive at all. Last, but not least, all potential survey participants could choose between two *modes of participation*: paper-based or online.

The *likelihood of cooperation* was measured by counting the total number of surveys that were answered by the survey respondents, following the American Association for Public Opinion Research 4 formula response rate classification. Hence, every filled-in questionnaire, on paper or via email, was marked as cooperative behavior, independently of whether respondents answered all or only a part of the questions. The main reason for this decision is that the MIFARE data do not distinguish between fully and partly filled-out questionnaires. Respondents who filled out the questionnaire twice, for example, online and on paper or both, in German and in their mother tongue, were counted only once.

Municipalities provided the MIFARE research team with information on the survey participants’ birth year, country of birth, gender, and residence. Participants’ country of origin was therefore identified as the country of birth. Given the further information provided by the municipalities, we can control for survey participants’ country of origin, gender, and size of municipality: (a) ≤50k inhabitants, (b) 50–100k inhabitants, (c) >100k<500k inhabitants, and (d) >500k inhabitants. Studies have found differences in contact and cooperation rates between migrant groups (Kappelhof, 2014). Similarly, gender affects both, likelihood of contact and cooperation. Whereas women generally are more likely to cooperate in survey research, this seems less to be the case for older migrant women (Sin, 2004; Wagner et al., 2019). Last, but not least, community size has been found to influence potential participants’ contact and cooperation rate, also among migrant groups (Kappelhof, 2014).

### Method

The MIFARE team contacted 12,660 migrants of whom 3,074 were 50 years or older. In the following, we present descriptive as well as analytical results for the likelihood of contact and the likelihood of cooperation among older migrants. After presenting the descriptives of the main variables by contact and cooperation, we continue with logistic regression estimations to examine the effects of age on the likelihood of contact, cooperation, and mode choice, controlling for gender, origin country, and municipality size in the sample population. For each outcome variable (contact, cooperation, mode choice), we first estimate the main effect of age (Model A.1, B.1, and C.1). In addition, we conduct several robustness checks testing whether the age effect is stable across gender (Model A.2, B.2, C.2) and migrant groups (Model A.3, B.3, C.3). Last, we examine the main effect of incentives on cooperation and its interaction with age (Model B.4, B.5). We thereby present the beta regression coefficient, the robust standard error, the *z*-value, the *p* value indicating the significance level, and finally the odds ratios (ORs) that indicate the probability of an event in relation to the probability of a nonevent. A value above 1 thereby indicates higher odds of an event occurring; a value below 1 indicates lower odds of an event occurring. In the following, all results will be interpreted in terms of the ORs.

Also, for every model, we indicate the model fit with the Wald Chi^2^ test, testing whether the given model is a significant improvement of the null model. Degrees of freedom are presented in square brackets.

## Results

### Descriptives

In a first step, we acquired a detailed overview of the contact and cooperation behavior of nine different migrant groups in Germany, comparing migrants aged 50 and older with younger migrants (Supplementary Table A1). We first look at the contact rate, the percentage of the sampling frame that the researchers were able to contact. Across all migrant groups and age groups, the contact rate was very high, usually above 90%. Generally, the results suggest that migrants of the age of 50 and older show a higher contact rate than younger migrants. Among those whom the researchers were able to contact, we look at the cooperation rate, the willingness, and ability to participate in the survey. On average 21% of all migrants participated in the survey and in the aggregate we do not see a difference between younger and older migrants in their cooperation behavior. However, looking at the different origin groups, we find that age indeed matters and that the effect varies depending on the country of origin. The cooperation rate is higher among older migrants from the United States, United Kingdom, Japan, and Romania. This is most visible among migrants from the United Kingdom, where around 25% of younger migrants participated in the survey compared to 38% among older migrants. However, among migrants from China, Russia, and Turkey, older migrants show lower cooperation rates. This suggests that when surveying older migrants, their national background has to be taken into account. Lastly, we look at the distribution of incentives and choice of mode among those migrants who responded to the survey. Among older migrants from China and Turkey, 32% and 29%, respectively, responded to the survey without receiving any incentive.

Before being able to make final conclusions, we apply multivariate analyses to examine cooperation behavior by comparing migrants who responded to the survey with migrants who did not respond. This finding already hints toward the direction that incentives are not equally important for all migrant groups and, equally interestingly, for all age groups. Also, most older migrants prefer participating in the survey using the paper version of the questionnaire.

### Likelihood of Contact

Table 1 presents the odds ratio of contact for migrants of 50 years of age and older, compared to younger migrants. We hypothesized that older migrants are less mobile than younger migrants and therefore easier to reach for survey participation. Indeed, we see that older migrants are significantly more likely to be contacted than migrants below the age of 50 (Model A.1, OR = 2.113, *p* < .001), which supports our first hypothesis (H1).

**Table 1.** Results Derived From Logistic Regressions Estimating Age Differences in Contact Probability

Variables	Model A.1					Model A.2					Model A.3				
	Coef.	*SE*	*z*	*p* > *z*	OR	Coef.	*SE*	*z*	*p* > *z*	OR	Coef.	*SE*	*z*	*p* > z	OR
Age ≥50	0.748	0.138	5.420	.000	2.113	0.574	0.167	3.440	.001	1.776	0.611	0.299	2.050	.041	1.842
Gender: Female	0.352	0.087	4.040	.000	1.422	0.301	0.092	3.280	.001	1.351	0.353	0.087	4.040	.000	1.423
Interaction Age × Gender						0.501	0.298	1.680	.093	1.651					
Origin country: USA (= ref.)															
UK	−0.075	0.159	−0.470	.636	0.927	−0.072	0.159	−0.450	.652	0.931	−0.099	0.174	−0.570	.568	0.906
China	−0.542	0.151	−3.600	.000	0.581	−0.541	0.151	−3.590	.000	0.582	−0.568	0.160	−3.550	.000	0.567
Japan	0.301	0.158	1.900	.057	1.352	0.300	0.158	1.890	.058	1.349	0.303	0.170	1.790	.074	1.355
Poland	0.562	0.222	2.530	.011	1.754	0.554	0.223	2.490	.013	1.741	0.543	0.243	2.230	.026	1.722
Romania	−0.536	0.222	−2.420	.016	0.585	−0.541	0.222	−2.430	.015	0.582	−0.568	0.231	−2.460	.014	0.567
Russia	1.878	0.408	4.610	.000	6.538	1.877	0.407	4.610	.000	6.531	1.681	0.412	4.080	.000	5.372
Spain	−0.367	0.144	−2.540	.011	0.693	−0.371	0.144	−2.570	.010	0.690	−0.384	0.154	−2.500	.012	0.681
Turkish	1.803	0.312	5.770	.000	6.066	1.802	0.312	5.770	.000	6.059	1.654	0.329	5.030	.000	5.229
Interaction Age × Origin country															
≥50 years × USA (= ref.)															
≥50 years × UK											0.156	0.431	0.360	.717	1.169
≥50 years × China											0.210	0.527	0.400	.690	1.234
≥50 years × Japan											−0.090	0.468	−0.190	.847	0.914
≥50 years × Poland											0.117	0.580	0.200	.840	1.125
≥50 years × Romania											0.208	0.510	0.410	.684	1.231
≥50 years × Russia											—	—	—	—	—
≥50 years × Spain											0.079	0.480	0.160	.870	1.082
≥50 years × Turkey											1.096	1.089	1.010	.314	2.993
Municipality size: <50k (= ref.)															
50–100k	−0.693	0.518	−1.340	.181	0.500	−0.691	0.518	−1.330	.183	0.501	−0.684	0.520	−1.320	.188	0.505
>100–500k	−1.215	0.382	−3.180	.001	0.297	−1.216	0.382	−3.180	.001	0.296	−1.208	0.386	−3.130	.002	0.299
>500k	−2.062	0.382	−5.400	.000	0.127	−2.061	0.382	−5.400	.000	0.127	−2.058	0.385	−5.340	.000	0.128
Model fit (Wald Chi ^2^ [13, 14, 20])	312.490					300.84					283.89				
Prop > Chi^2^	0.000					0.000					0.000				
*N*	12,660					12,660					12,660				

### Likelihood of Cooperation

Table 2 presents that older migrants are not only more likely to be contacted; among those who received an invitation, older migrants are also more likely to cooperate in the survey (Model B.1, OR = 1.185, *p* = .001). This finding goes against our hypothesis H2a, where we assumed lower cooperation rates among older migrants and supports H2b.

**Table 2.** Results Derived From Logistic Regressions Estimating Age Differences in Cooperation Probability

Variables	Model B.1					Model B.2					Model B.3				
	Coef.	*SE*	*z*	*p* > *z*	OR	Coef.	*SE*	*z*	*p* > *z*	OR	Coef.	*SE*	*z*	*p* > *z*	OR
Age ≥50	0.170	0.053	3.200	.001	1.185	0.356	0.074	4.790	.000	1.428	0.361	0.149	2.420	.016	1.434
Gender: Female	0.189	0.046	4.140	.000	1.208	0.281	0.053	5.310	.000	1.324	0.191	0.046	4.180	.000	1.211
Interaction															
Age × Gender						−0.366	0.104	−3.510	.000	0.694					
Origin country: USA (=ref.)															
UK	0.256	0.096	2.670	.008	1.292	0.252	0.096	2.630	.009	1.287	0.168	0.116	1.450	.147	1.183
China	0.250	0.100	2.500	.012	1.284	0.251	0.100	2.500	.012	1.285	0.371	0.111	3.340	.001	1.449
Japan	−0.011	0.090	−0.120	.905	0.989	−0.010	0.090	−0.110	.915	0.990	0.003	0.104	0.030	.979	1.003
Poland	−0.548	0.112	−4.900	.000	0.578	−0.543	0.112	−4.850	.000	0.581	−0.497	0.130	−3.830	.000	0.608
Romania	−0.404	0.123	−3.280	.001	0.668	−0.401	0.123	−3.260	.001	0.669	−0.415	0.142	−2.920	.003	0.660
Russia	−0.391	0.115	−3.400	.001	0.676	−0.391	0.115	−3.400	.001	0.676	−0.241	0.129	−1.870	.062	0.786
Spain	0.252	0.094	2.690	.007	1.287	0.260	0.094	2.770	.006	1.297	0.317	0.106	3.000	.003	1.373
Turkish	−1.014	0.115	−8.850	.000	0.363	−1.013	0.115	−8.840	.000	0.363	−0.818	0.129	−6.320	.000	0.441
Interaction															
Age × Origin country															
≥50 years × USA (= ref.)															
≥50 years × UK											0.257	0.210	1.220	.222	1.292
≥50 years × China											−0.876	0.306	−2.870	.004	0.417
≥50 years × Japan											0.014	0.212	0.070	.946	1.014
≥50 years × Poland											−0.155	0.209	−0.740	.458	0.857
≥50 years × Romania											0.027	0.222	0.120	.902	1.028
≥50 years × Russia											−0.506	0.209	−2.430	.015	0.603
≥50 years × Spain											−0.288	0.249	−1.160	.248	0.750
≥50 years × Turkey											−0.595	0.216	−2.760	.006	0.552
Incentives: None (= ref.)															
Conditional															
Unconditional															
Conditional + Unconditional															
Interaction															
Age × Incentives															
≥50 × No incentive (= ref.)															
≥50 × Conditional															
≥50 × Unconditional															
≥50 × Conditional + Unconditional															
Municipality size: <50k (= ref.)															
50–100k	0.103	0.118	0.870	.383	1.109	0.103	0.118	0.870	.383	1.109	0.103	0.119	0.870	.384	1.109
>100–500k	0.081	0.093	0.870	.385	1.084	0.079	0.093	0.860	.391	1.083	0.082	0.093	0.880	.378	1.085
>500k	−0.022	0.102	−0.210	.832	0.979	−0.025	0.102	−0.250	.804	0.975	−0.007	0.102	−0.060	.948	0.993
Model fit (Wald Chi^2^ [13, 14, 21, 16, 19])	280.51					293.83					319.70				
Prop > Chi^2^	0.000					0.000					0.000				
*N*	12,097					12,097					12,097				
	Model B.4					Model B.5									
Age ≥50	0.173	0.053	3.240	.001	1.188	0.138	0.124	1.110	.266	1.148					
Gender: Female	0.184	0.046	4.050	.000	1.203	0.182	0.046	4.000	.000	1.200					
Interaction															
Age × Gender															
Origin country: USA (= ref.)															
UK	0.257	0.096	2.680	.007	1.293	0.258	0.096	2.680	.007	1.294					
China	0.253	0.100	2.530	.011	1.288	0.254	0.100	2.540	.011	1.290					
Japan	−0.008	0.090	−0.090	.925	0.992	−0.006	0.090	−0.070	.943	0.994					
Poland	−0.549	0.112	−4.910	.000	0.578	−0.552	0.112	−4.930	.000	0.576					
Romania	−0.407	0.123	−3.300	.001	0.666	−0.407	0.123	−3.300	.001	0.666					
Russia	−0.391	0.115	−3.400	.001	0.676	−0.391	0.115	−3.390	.001	0.677					
Spain	0.256	0.094	2.740	.006	1.292	0.259	0.094	2.770	.006	1.296					
Turkish	−1.016	0.115	−8.860	.000	0.362	−1.017	0.115	−8.870	.000	0.362					
Interaction															
Age × Origin country															
≥50 years × USA (=ref.)															
≥50 years × UK															
≥50 years × China															
≥50 years × Japan															
≥50 years × Poland															
≥50 years × Romania															
≥50 years × Russia															
≥50 years × Spain															
≥50 years × Turkey															
Incentives: None (= ref.)															
Conditional	0.027	0.070	0.390	.695	1.028	−0.028	0.082	−0.340	.732	0.972					
Unconditional	0.275	0.068	4.040	.000	1.316	0.310	0.078	3.970	.000	1.363					
Conditional + Unconditional	0.278	0.068	4.100	.000	1.321	0.260	0.078	3.330	.001	1.297					
Interaction															
Age × Incentives															
≥50 × No incentive (= ref.)															
≥50 × Conditional						0.208	0.160	1.300	.195	1.231					
≥50 × Unconditional						−0.141	0.159	−0.890	.376	0.869					
≥50 × Conditional + Unconditional						0.075	0.158	0.480	.635	1.078					
Municipality size: <50k (= ref.)															
50–100k	0.103	0.119	0.860	.388	1.108	0.101	0.119	0.850	.393	1.107					
>100–500k	0.081	0.093	0.870	.384	1.084	0.080	0.093	0.860	.388	1.084					
>500k	−0.024	0.103	−0.230	.818	0.977	−0.027	0.103	−0.260	.795	0.974					
Model fit (Wald Chi^2^ [13, 14, 21, 16, 19])	304.84					310.67									
Prop > Chi^2^	0.000					0.000									
*N*	12,097					12,097									

Model B.4 shows the odds ratio of participating in the survey depending on the incentive respondents received. We see that in comparison with migrants who did not receive an incentive at all, those migrants who received the unconditional incentive (mobile screen cleaner) and those who received the unconditional and the conditional incentive (mobile screen cleaner + 10€ voucher) were significantly more likely to participate in the survey (OR_Unconditional_ = 1.136, *p* = .001; OR_Conditional + Unconditional_ = 1.321, *p* = .001). Interestingly, migrants who were offered only a conditional incentive did not differ in their participation behavior from those who received no incentive at all (OR = 1.028, *p* = .695). In addition, the effects of these incentive treatments do not vary between older and younger migrants (Model B.5: OR_Age × Conditional_ = 1.231, *p* = .195; OR_Age × Unconditional_ = 0.869, *p* = .376; OR_Age × Conditional + Unconditional_ = 1.078, *p* = .635). Hence, neither Hypothesis 3a nor 3b was supported.

In the last step, we examined whether older migrants have different preferences with regard to the choice of mode than younger migrants (Table 3). All potential survey participants could choose to fill out the survey either on paper or online. Results from Table 3 show that, as expected, older migrants have a significantly stronger preference to fill out the survey on paper than migrants below the age of 50 (OR = 1.965, *p* = .000), supporting the fourth hypothesis (H4).

**Table 3.** Results Derived From Logistic Regressions Estimating Age Differences in Mode Choice

Variables	Model C.1					Model C.2					Model C.3				
	Coef.	*SE*	*z*	*p* > *z*	OR	Coef.	*SE*	*z*	*p* > *z*	OR	Coef.	*SE*	*z*	*p* > *z*	OR
Age ≥50	0.675	0.120	5.640	.000	1.965	0.720	0.154	4.670	.000	2.055	0.801	0.288	2.780	.005	2.228
Gender: Female	0.528	0.095	5.560	.000	1.696	0.548	0.105	5.210	.000	1.730	0.537	0.095	5.640	.000	1.711
Interaction Age × Gender						−0.108	0.237	−0.460	0.648	0.898					
Origin country: USA (= ref.)															
UK	0.121	0.178	0.680	.497	1.129	0.119	0.178	0.670	.505	1.126	0.318	0.213	1.490	.136	1.374
China	0.310	0.190	1.630	.102	1.363	0.312	0.190	1.640	.101	1.365	0.353	0.205	1.720	.085	1.423
Japan	0.560	0.178	3.140	.002	1.751	0.562	0.178	3.160	.002	1.755	0.693	0.201	3.450	.001	2.000
Poland	1.138	0.260	4.380	.000	3.122	1.141	0.260	4.390	.000	3.129	1.125	0.285	3.950	.000	3.079
Romania	0.439	0.254	1.720	.085	1.550	0.440	0.255	1.730	.084	1.553	0.509	0.289	1.760	.078	1.663
Russia	0.527	0.229	2.300	.021	1.693	0.527	0.229	2.300	.021	1.693	0.396	0.247	1.600	.109	1.486
Spain	0.235	0.174	1.350	.177	1.265	0.237	0.174	1.360	.173	1.267	0.277	0.192	1.450	.148	1.320
Turkish	0.714	0.233	3.060	.002	2.042	0.714	0.233	3.060	.002	2.041	0.653	0.260	2.510	.012	1.921
Interaction Age × Origin country															
≥50 years × USA (= ref.)															
≥50 years × UK											−0.591	0.386	−1.530	.126	0.554
≥50 years × China											−0.314	0.625	−0.500	.616	0.731
≥50 years × Japan											−0.699	0.427	−1.640	.102	0.497
≥50 years × Poland											0.159	0.552	0.290	.773	1.172
≥50 years × Romania											−0.187	0.466	−0.400	.689	0.830
≥50 years × Russia											1.265	0.601	2.110	.035	3.543
≥50 years × Spain											−0.229	0.502	−0.460	.649	0.796
≥50 years × Turkey											0.454	0.555	0.820	.413	1.574
Incentives: None (= ref.)															
Conditional	−0.002	0.149	−0.010	.990	0.998	−0.001	0.149	0.000	.997	0.999	0.007	0.149	0.050	.962	1.007
Unconditional	−0.232	0.140	−1.660	.098	0.793	−0.232	0.140	−1.660	.098	0.793	−0.221	0.141	−1.570	.116	0.802
Conditional + Unconditional	−0.180	0.142	−1.260	.206	0.836	−0.179	0.142	−1.260	.208	0.836	−0.176	0.143	−1.230	.220	0.839
Municipality size: <50k (= ref.)															
50–100k	−0.027	0.267	−0.100	.919	0.973	−0.027	0.267	−0.100	.920	0.973	−0.045	0.275	−0.160	.870	0.956
>100–500k	0.132	0.221	0.600	.550	1.141	0.131	0.221	0.590	.553	1.140	0.159	0.228	0.700	.484	1.173
>500k	−0.104	0.233	−0.450	.655	0.901	-0.105	0.233	−0.450	.653	0.901	−0.074	0.240	−0.310	.756	0.928
Model fit (Wald Chi^2^ [16, 17, 24])	122.640					124.240					126.270				
Prop > Chi^2^	0.000					0.000					0.000				
*N*	2,499					2,499					2,499				

### Robustness Checks

We also conducted several robustness checks to test the generalizability of our results. For each of our three main models (contact, cooperation, and mode), we therefore conducted further exploratory analyses and estimated an interaction effect of age and gender as well as age and country of origin. For likelihood of contact (Table 1), we observe that the age effect is significantly stronger for female migrants than for male migrants (Model A.2, OR = 1.422, *p* = .000). Interestingly, the age effect does not vary significantly between different migrant groups.

For our second aspect, cooperation, we tested whether the age effect on survey cooperation remains stable across origin groups (Table 2). We therefore estimated an interaction effect between migrants’ age group and their country of origin. In Model B.3, we see that the age effect does not vary for most migrant groups. Only for migrants from China, Russia, and Turkey, older age decreases the likelihood of participating in the survey (OR_China_ = 0.417, *p* = .004; OR_Russia_ = 0.603, *p* = .015; OR_Turkey_ = 0.552, *p* < .006). We also find that female migrants are significantly more likely to participate in the MIFARE survey than male migrants (Model B.1, OR = 1.208, *p* = .000). However, this is less so among older migrants. The odds that female migrants are more likely to participate in a survey than male migrants are lower among younger than among older migrants (Model B.2, OR = 0.694, *p* = .000).

Finally, we tested whether the age effect on mode choice holds equally for male and female migrants and across different origin groups (Table 3). Whereas female migrants are significantly more likely to fill out the survey on paper than male migrants (Model C.1, OR = 1.696, *p* = .000), the interaction effect between age and gender is not significant (Model C.2, OR = 0.898, *p* = .648). In Model C.3, we also estimated the interaction effect between age and origin group and find that only for migrants from Russia the age effect is significantly stronger compared to migrants from the United States (OR = 3.543, *p* = .035).

## Discussion

This study contributes to the existing research on nonresponse among migrants by examining survey design among migrants through the lens of age. Using data from the MIFARE survey, we study both the likelihood of contact and the likelihood of cooperation, comparing older first-generation migrants (older than the age of 50) from nine different origin groups with younger first-generation migrants (aged 16–49). Due to the demographic change, older migrants become an increasingly important group to study. However, surveying this population brings specific challenges, because older migrants often differ from younger migrants in terms of mobility, language barriers, attitudes toward survey research, and connectedness to the host country (Sin, 2004). We therefore look at two main factors in survey research, namely contact and cooperation. In addition, we examine the extent to which older migrants respond to different types of incentives and whether they prefer participation on paper or online.

Results indicate the following: First, older migrants are more likely to be contacted than younger migrants. A likely explanation for this finding is the decreasing mobility that comes with age. Due to changing living situations such as finding new employment or starting a family, younger migrants are comparatively more mobile, particularly within the first years after migration (Blohm & Diehl, 2001; Kappelhof, 2014). This is less the case for older migrants, which might be a plausible explanation for our finding. We also find that the age effect is particularly strong for female migrants. This suggests that older female migrants are less mobile than older male migrants and are therefore easier to be contacted. Interestingly, the likelihood of contacting older migrants does not vary among the nine migrant groups.

Second, we found older migrants to be more likely than younger migrants to participate in the survey, which goes against our second hypothesis. Previous studies generally found lower participation rates among older people than among younger people (Deding et al., 2008; Font & Méndez, 2013; Gaertner et al., 2016; Motel-Klingebiel et al., 2019). However, these studies investigate migrants older than the age of 65, whereas we specifically compare migrants of the age of 50 and older to migrants of the age 16–49. It is likely that age has a curvilinear effect on participation rates. Migrants of the age of 50 might be generally healthier and more likely to be employed compared with their cohorts older than the age of 65 (Kotwal, 2010). Hence, the assumed mechanisms described above might only apply to the very old.

Third, we found that older and younger migrants did not differ in their reactions to the different types of incentives. Among both age groups, we see the highest response rate among those migrants who received (a) only the unconditional and (b) the unconditional plus the conditional incentives. The use of only the conditional incentive had no effect on the response rate. This is a relevant finding because it suggests that costly monetary conditional incentives might not have the effect on the response rate that is assumed. Fourth, we found, not surprisingly, that older migrants have a strong preference for filling out the questionnaire on paper, rather than online. This is in line with previous research among general populations (Miller et al., 2002). In addition, we find that the preference for paper-based questionnaires is particularly strong for older female migrants. This supports previous research finding that men are on average more likely to respond to the web-based survey than women (Bech & Kristensen, 2009; Couper, 2000; McDonald & Adam, 2003).

Of course, this study also contains limitations. As discussed already, we might have overestimated the contact rate among both groups, older and younger migrants, because we do not know whether some of the potential survey participants might have been on vacation or a longer trip to their home country. In these cases, the survey invitation reached the house, but not the potential survey participant. In this study, we would, by definition, interpret these cases as being successfully contacted, hence, overestimating our contact rate. Also, given that the MIFARE survey was not targeted to older migrants, little can be said about optimizing the sampling frame and factors reducing the risk of coverage error. Previous research suggests using records from, for example, family practitioners or pension records to achieve a representative sampling frame (Sin, 2004). Also, the data analyzed for this study refer to the German context only and results should be externalized to another context with caution. For example, old-age poverty or old-age mobility might vary between countries, thereby affecting the contact and cooperation rate among older migrants. In addition, information on potential respondents’ socioeconomic characteristics, which most likely influence their likelihood of being contacted and to cooperate, is missing. Particularly for older migrants, their health status is likely to influence their willingness to participate. This is generally a problem in survey research as we know too little about people who do not participate in the survey. Last, but not least, this study does not provide a comparison between older migrants and older nonmigrants. We know from previous research that migrants and nonmigrants differ significantly in their response behavior (Feskens et al., 2006; Haan et al., 2014). We therefore suggest that future research examines the interaction between age and ethnic background to acquire more insight into response rates among older migrants. Still, this study provides useful information about surveying older first-generation migrants. Offering unconditional incentives and surveys on paper are likely to increase response rates among older migrants. Future research should examine further why the likelihood of contact and cooperation among first-generation migrants differs between men and women and among different origin groups.

## Supplementary Material

gnac017_suppl_Supplementary_Material
