# Supplementary material for: Survey Research Among Older Migrants: Age-Related Differences in Contact and Cooperation
Source: Gerontologist. 2022 Jan 29;62(6):842–54. doi: 10.1093/geront/gnac017 (PMC9295202; doi:10.1093/geront/gnac017)
Supplement: gnac017_suppl_Supplementary_Material [file gnac017_suppl_supplementary_material.docx]

*The Gerontologist* Online Supplementary Material: Seibel, Verena, & Haan, Marieke. Survey research among older migrants: Age-related differences in contact and cooperation.

| Supplementary Table A1: Descriptives (percentages) of main variables by age group and migrant origin country | | | | | | | | | | | | | | | | | | | | |
| --- | --- | --- | --- | --- | --- | --- | --- | --- | --- | --- | --- | --- | --- | --- | --- | --- | --- | --- | --- | --- |
|  | **All** | | **USA** | | **UK** | | **China** | | **Japan** | | **Poland** | | **Romania** | | **Russia** | | **Spain** | | **Turkey** | |
| Age (in years)/ Main variables | <  50 | >= 50 | <  50 | >= 50 | <  50 | >= 50 | <  50 | >= 50 | <  50 | >= 50 | <  50 | >= 50 | <  50 | >= 50 | <  50 | >= 50 | <  50 | >= 50 | <  50 | >= 50 |
| **Contact/Sampling frame** | 94% | 98% | 92% | 96% | 91% | 96% | 89% | 94% | 94% | 97% | 98% | 99% | 94% | 98% | 99% | 100% | 89% | 94% | 99% | 100% |
| **Cooperation/Sample** | 21% | 21% | 22% | 29% | 25% | 38% | 30% | 20% | 22% | 30% | 15% | 18% | 16% | 22% | 20% | 18% | 28% | 30% | 12% | 10% |
| **Incentives/Response** |  |  |  |  |  |  |  |  |  |  |  |  |  |  |  |  |  |  |  |  |
| No incentive | 18% | 17% | 14% | 10%% | 21% | 16% | 18% | 32% | 17% | 18% | 15% | 16% | 21% | 14% | 18% | 21% | 18% | 10% | 22% | 28% |
| Conditional | 23% | 27% | 21% | 30% | 22% | 27% | 23% | 32% | 20% | 26% | 27% | 28% | 26% | 22% | 24% | 26% | 22% | 29% | 20% | 29% |
| Unconditional | 30% | 27% | 35% | 30% | 25% | 23% | 31% | 26% | 33% | 30% | 27% | 29% | 29% | 32% | 30% | 23% | 28% | 32% | 31% | 20% |
| Conditional+Unconditional | 29% | 29% | 30% | 29% | 32% | 33% | 29% | 11% | 30% | 26% | 31% | 28% | 24% | 32% | 27% | 30% | 32% | 29% | 26% | 23% |
| **Mode Choice/Response** |  |  |  |  |  |  |  |  |  |  |  |  |  |  |  |  |  |  |  |  |
| on paper | 71% | 82% | 60% | 75% | 66% | 68% | 69% | 79% | 76% | 78% | 84% | 93% | 74% | 83% | 72% | 95% | 66% | 78% | 75% | 91% |
| online | 29% | 18% | 40% | 25% | 34% | 32% | 31% | 21% | 24% | 22% | 16% | 7% | 26% | 17% | 28% | 5% | 34% | 22% | 25% | 9% |
| N Sampling Frame | 9,592 | 3,068 | 992 | 320 | 806 | 305 | 915 | 101 | 1,331 | 266 | 1,086 | 469 | 788 | 341 | 1,177 | 440 | 1,132 | 143 | 1,365 | 683 |
| N Sample | 9,058 | 3,005 | 916 | 306 | 737 | 292 | 811 | 95 | 1,257 | 257 | 1,062 | 464 | 742 | 334 | 1,169 | 440 | 1,010 | 135 | 1,354 | 682 |
| N Response | 1,891 | 642 | 205 | 89 | 186 | 111 | 244 | 19 | 287 | 77 | 166 | 87 | 124 | 76 | 233 | 77 | 287 | 41 | 159 | 65 |
|  |  |  |  |  |  |  |  |  |  |  |  |  |  |  |  |  |  |  |  |  |
